# Genome-resolved analysis of colonization factor repertoires reveals ecological stratification in cervid gut microbiomes

**DOI:** 10.3389/fmicb.2026.1929306

**Published:** 2026-08-27

**Authors:** Hao Ni, Qing-Long Gong, Jian-Ming Li, Fei Liu, Xue Leng, Yu-Hao Song, Jing Jiang, Zhong-Yuan Li, Ying Li, Yu-Zhe Sun

**Affiliations:** 1 College of Life Sciences, Changchun Sci-Tech University, Changchun, Jilin, China; 2 College of Food and Pharmaceutical Engineering, Wuzhou University, Wuzhou, China; 3 College of Animal Science and Veterinary Medicine, Heilongjiang Bayi Agricultural University, Daqing, Heilongjiang, China; 4 College of Veterinary Medicine, Jilin Agricultural University, Changchun, Jilin, China; 5 College of Chinese Medicine Materials, Jilin Agricultural University, Changchun, Jilin, China; 6 Guangxi Key Laboratory of Drug Discovery and Optimization, Guangxi Engineering Research Center for Pharmaceutical Molecular Screening and Druggability Evaluation, Key Laboratory of Medical Biotechnology and Translational Medicine, School of Pharmacy, Guilin Medical University, Guilin, China; 7 College of Veterinary Medicine, Qingdao Agricultural University, Qingdao, Shandong, China

**Keywords:** Cervidae, colonization factors, ecological stratification, gut microbiome, metagenomics

## Abstract

**Introduction:**

Colonization factors (CFs) are important microbial traits associated with persistence and host adaptation in the gut, yet their large-scale organization in cervid gut microbiomes remains unclear.

**Methods:**

A total of 3,311 non-redundant high-quality metagenome-assembled genomes (MAGs), derived from 688 cervid gut metagenomic samples across 15 publicly available projects and one in-house dataset, were analyzed. CF-associated genes were identified by comparison against the GHA CF database, and CF repertoires were characterized at genome, host-species, and gastrointestinal-segment levels.

**Results:**

A total of 138,729 CF-associated genes spanning 71 CF families were identified. MAGs from Cervinae contained richer CF repertoires than those from Caprinae, and CF47 (Peptidase_C69), CF24_29 (QueH), and CF18 (Glycos_transf_2) were among the most prevalent families. CF repertoires were strongly structured by taxonomy, showed a moderate association with bacterial phylogenetic distance, and formed two recurrent genome-level configurations with distinct KEGG functional profiles. Integration of sample metadata further revealed differentiation of CF repertoires across host species and gastrointestinal segments, representing the major ecological dimensions examined in this study. Segment-associated CF variation was accompanied by redistribution of broader functional profiles, including enrichment of carbohydrate and lipid metabolism in the jejunum, membrane transport in the ileum, xenobiotics biodegradation in the cecum, and environmental adaptation in the rumen.

**Discussion:**

These findings provide a genome-resolved view of CF repertoire organization in cervid gut microbiomes and demonstrate that colonization-associated functions are structured across microbial lineages and ecological contexts. This study highlights the importance of considering microbial taxonomy and host-associated environments when interpreting the distribution of CF repertoires in mammalian gut ecosystems.

## Introduction

Stable microbial colonization is a prerequisite for long-term persistence in the gut and underlies many aspects of host-microbiome interactions (Parker et al., 2018; Pirr and Viemann, 2020). In this study, colonization factors (CFs) were operationally defined according to the Gut Host Adhesion (GHA) database framework. Under this framework, CFs comprise database-defined colonization-associated genes involved in functions including nutrient acquisition, environmental adaptation, transport, adhesion, and interactions with host-associated surfaces (Lee et al., 2013; Lin et al., 2024; Muramatsu and Winter, 2024). Accordingly, the term “CF” is used here to denote GHA-defined genes identified through sequence homology, rather than exclusively experimentally validated determinants of microbial colonization. However, despite their biological importance, CFs have been studied mainly in individual organisms or limited experimental systems, and their large-scale organization across complex gut microbiomes remains insufficiently resolved.

Cervid gut microbiomes are shaped by multiple layers of host-associated heterogeneity. Cervids are particularly informative for studying CF organization because their ecological and host-lineage diversity is superimposed on the strongly compartmentalized digestive system of ruminants, allowing colonization-associated functions to be compared across both host and gastrointestinal contexts. The availability of metagenomic datasets spanning multiple cervid hosts and gastrointestinal regions further enables these patterns to be examined within a comparative genome-resolved framework. Previous studies have shown that gut microbial communities can differ substantially across host species, among closely related host lineages, and between broader ruminant groups, with corresponding shifts in both taxonomic composition and functional potential (Glendinning et al., 2021; Rojas et al., 2021; Sun et al., 2025, 2026a). A second source of variation lies within the gastrointestinal tract itself, where individual compartments differ in physicochemical conditions, substrate availability, host secretions, and local microbial interaction structure (Cholewińska et al., 2020; Yang et al., 2025). Such heterogeneity is likely to impose distinct selective pressures on microbial persistence traits across host backgrounds and intestinal niches (Adak and Khan, 2019; Pereira and Berry, 2017). CF repertoires therefore are unlikely to be maintained as a uniform functional background and may instead vary systematically across host species and gut compartments.

However, documenting ecological differentiation alone is not sufficient to explain how colonization-related functions are organized. Differences across hosts or gut regions may arise through several non-exclusive processes, including taxonomic turnover, phylogenetic constraint, and recurrent combinations of persistence-related traits embedded within broader genomic backgrounds (Parfrey et al., 2018; Shafquat et al., 2014). A more informative framework therefore requires asking how CF repertoires are distributed across microbial genomes, whether they show strong taxonomic and phylogenetic structuring, whether they form recurrent genome-level configurations, and how these configurations vary across host-associated ecological contexts (Martiny et al., 2015; Moeller et al., 2023). Addressing these questions is necessary for interpreting CF variation not simply as a collection of isolated functional shifts, but as an organizational property of host-associated microbiomes.

A genome-resolved comparative framework enables colonization-related functions to be evaluated simultaneously across microbial lineage, host background, and intestinal niche (Beghini et al., 2021; Zhang Y. et al., 2026). This scale of analysis is important for distinguishing variation associated primarily with taxonomic replacement from variation reflecting broader restructuring of colonization-related functional repertoires.

Here, we hypothesized that CF repertoires are hierarchically structured rather than randomly distributed across cervid gut microbiomes. Specifically, we expected microbial taxonomy and phylogeny to constrain genome-level CF organization, while host species and gastrointestinal segment would drive ecological differentiation in CF composition. We further expected these patterns to be expressed through recurrent genome-level CF configurations with distinct functional backgrounds. In this framework, we use “CF organization” to describe the distribution and configuration of CF repertoires across microbial genomes, “ecological differentiation” to describe variation among host species and gastrointestinal segments, and “ecological stratification” to describe the combined hierarchical pattern generated across genomic, phylogenetic, and ecological levels. To test this hypothesis, our primary objectives were to characterize the taxonomic and phylogenetic structure of CF repertoires, identify recurrent genome-level configurations, and evaluate their differentiation across host species and gastrointestinal segments. The Cervinae–Caprinae comparison was included to provide broader ruminant context, whereas KEGG-based profiling was used to interpret the functional background associated with the observed CF patterns.

## Materials and methods

### Data collection

We analyzed 15,494 MAGs previously generated from 688 cervid gut metagenomic samples in our earlier study. The dataset comprised 15 publicly available metagenomic projects and one in-house dataset and was obtained from Figshare^1^ (Sun et al., 2026b). In the previous study, raw-read quality control, host-read removal, metagenomic assembly, and genome binning were performed to generate the initial MAG set. In the present study, these 15,494 MAGs were used as the starting dataset for genome quality filtering, dereplication, taxonomic assignment, gene prediction, and downstream CF analyses. For transparency and reproducibility, we summarize below the assembly and binning pipeline and parameter settings used to generate the MAGs in the earlier work. Detailed information for all 688 samples is provided in Supplementary Table 1.

### Reference sequence retrieval for CF identification

Colonization factor reference sequences were retrieved from the GHA database (accessed December 2025), yielding 27,096 amino acid sequences used as references for homology-based identification of CF genes.

### Genome-resolved metagenomic analysis

Raw reads were quality filtered using fastp (v0.20.1) (Chen et al., 2018). Host-derived reads were removed by mapping against host reference genomes using Bowtie2 (v2.5.4) (Langmead and Salzberg, 2012). Filtered reads were assembled with MEGAHIT (v1.2.9) (Li et al., 2015), and contigs were binned into MAGs using MetaBAT2 (v2.12.1) (Kang et al., 2019).

Metagenome-assembled genome quality was evaluated with CheckM2 (v1.1.0) (Chklovski et al., 2023), and high-quality MAGs were defined as those with completeness ≥ 90% and contamination ≤ 5% (Bowers et al., 2017). Redundant MAGs were dereplicated at 99% average nucleotide identity using dRep (v3.6.2) (Olm et al., 2017). Taxonomic assignments were obtained using GTDB-Tk (v2.3.2) (Chaumeil et al., 2019). Protein-coding genes were predicted with Prodigal (v2.6.3) (Hyatt et al., 2010), and the resulting protein sequences were used for downstream CF annotation.

### Phylogenomic reconstruction and visualization

A phylogenomic tree of the 3,311 non-redundant MAGs was reconstructed from the predicted protein sequences using PhyloPhlAn (v3.0.67). Conserved phylogenetic markers were identified and processed using the standard protein-based workflow implemented in PhyloPhlAn3, including marker alignment, alignment refinement, concatenation, and phylogenetic tree inference. GTDB-Tk taxonomic assignments and genome characteristics were subsequently incorporated as annotation layers, and the resulting tree was visualized using the Interactive Tree of Life (iTOL).

### Identification of CF

Predicted proteins from the non-redundant MAG set were searched against the GHA CF reference database using DIAMOND BLASTP (v2.1.11) (Altschul et al., 1997; Buchfink et al., 2015). CF candidates were defined as hits with an *E*-value ≤ 1 × 10^–10^ and both query and subject coverage ≥ 70%. For each query protein, the top-ranked hit based on bit score was retained as the CF assignment. Each predicted protein sequence was treated as an independent query during sequence alignment.

Colonization factor hits were mapped back to their source MAGs and linked to GTDB-Tk taxonomic annotations. To capture within-species variation in individual CF genes, a CF reference gene was considered present in a species when detected in at least one genome assigned to that species. To define a representative species-level CF repertoire and reduce the influence of sporadic genome-specific detections, a CF family was considered present only when detected in at least 50% of the genomes assigned to that species (Tettelin et al., 2008). The reference-gene criterion was used to summarize species-level gene occurrence, whereas the 50% family-level criterion was used to construct the species-level CF-family matrix for downstream repertoire analyses.

### Cross-subfamily comparison of CF repertoires

To place cervine CF repertoires in a broader ruminant context, a Caprinae metagenomic dataset was additionally included for cross-subfamily comparison. The Cervinae and Caprinae genome sets were processed independently using the same quality-control, assembly, binning, gene prediction, and CF annotation workflow. Within each dataset, MAGs meeting the quality thresholds of completeness ≥ 90% and contamination ≤ 5% were independently dereplicated at 99% average nucleotide identity. This resulted in 3,311 non-redundant Cervinae MAGs and 3,672 non-redundant Caprinae MAGs for genome-level CF comparison.

### Functional annotation of CF-associated proteins

To characterize the domain composition of CF-associated proteins, all CF-hit proteins were searched against the Pfam-A HMM database (release 37.4, accessed January 2026) using hmmscan from HMMER with the Pfam gathering threshold (–cut_ga) (Eddy, 2011; Mistry et al., 2021). Multiple domains per protein were permitted, and the domain with the lowest *E*-value was retained as the representative Pfam annotation for downstream analyses. KEGG Orthology (KO) assignments were obtained by aligning the same protein sequences against the KEGG database (Kanehisa et al., 2023).

### Clustering of CF repertoires

Genome-level CF presence–absence matrices were constructed to investigate structural patterns of CF repertoires. Pairwise Jaccard distances were calculated from the binary matrix to quantify dissimilarity among genome-level CF repertoires, and PAM clustering was applied to group genomes with similar CF repertoires. The Calinski–Harabasz index was used to determine the optimal cluster number.

For downstream analyses, each CF family was assigned to the genome cluster in which it showed the highest prevalence. This family-to-cluster mapping was then used to derive sample-level cluster-associated CF abundance profiles by summing the abundances of CF-associated genes belonging to families assigned to the same cluster. These values therefore represent abundance-weighted summaries of cluster-associated CF families within each metagenomic sample, rather than direct assignments of metagenomic samples to genome clusters. The resulting clusters were interpreted as recurrent structural configurations of CF repertoires based on similarity in genome-level presence–absence profiles, rather than as experimentally validated colonization strategies.

### Quantification of CF-associated gene abundance

A nucleotide gene catalog was constructed from the predicted coding sequences in FFN format. Quality-filtered paired-end reads were aligned to the catalog using Bowtie2 in end-to-end mode with the –fast preset. Gene-level read counts were obtained from the reported alignments, with one alignment retained for reads mapping equally well to multiple reference genes. Gene lengths were calculated using SeqKit. TPM values were calculated by dividing the read count of each gene by its sequence length and scaling the resulting length-normalized counts to one million within each sample. TPM values ≤ 10 were set to zero, and genes with zero abundance across all samples were excluded. The resulting TPM matrix was matched to the CF annotation table by gene identifier for downstream analyses.

### Host-species comparison of CF repertoires

For host-species comparisons, only samples that could be unambiguously assigned to four focal cervine species [*Cervus nippon* (Yan et al., 2022), *Cervus albirostris* (Li et al., 2025), *Cervus canadensis* (Li et al., 2025), and *Cervus elaphus* (Glendinning et al., 2021; Guo et al., 2024a,b)] were retained (*n* = 279) (Supplementary Table 2). CF-associated genes were extracted by matching gene identifiers to the CF annotation table, and interspecies differences were assessed using Bray–Curtis PCoA with PERMANOVA, Kruskal–Wallis tests of CF-gene richness and Shannon diversity, and cluster-level comparisons based on predefined CF assignments. To provide host evolutionary context for these interspecies comparisons, a BUSCO-based phylogeny was reconstructed for the four focal *Cervus* species, with *Moschus berezovskii* (Moschidae), *Bos taurus*, and *Capra hircus* included as outgroup taxa.

### Gut-segment comparison of CF repertoires

Segment-level comparisons were performed using 75 metagenomic samples from a single project (PRJNA779578) (Supplementary Table 3; Yan et al., 2022), including 15 samples each from the rumen, jejunum, ileum, cecum, and colon. CF-associated genes were extracted by matching gene identifiers to the CF annotation table. Segment-associated variation in CF-gene composition was visualized using Bray–Curtis PCoA, while differences in CF-gene richness and Shannon diversity were assessed using the Kruskal–Wallis test.

### Functional analyses for interpreting segment-associated CF differentiation

To further interpret segment-associated CF differences, CF-associated genes were grouped according to the predefined cluster assignments, and gene abundances were summed within each cluster to generate sample-level cluster profiles for inter-segment comparison. KEGG-based analyses were performed on the same 75 samples to characterize the functional context associated with segment-specific CF differentiation. Overall KEGG compositional differences were assessed using Bray–Curtis PCoA and PERMANOVA. For visualization of segment-associated functional variation, a subset of KEGG B-level categories was displayed to summarize the major functional patterns across gastrointestinal segments. This selection was limited to data presentation and did not affect the analysis of the complete KEGG profiles. Associations between KEGG categories and CF organization were evaluated using abundance-weighted Spearman correlations at the CF family level, with Benjamini–Hochberg correction applied for multiple testing.

### Statistical frameworks and data visualization

All statistical analyses and visualizations were performed in R (v4.5.0). Bray–Curtis dissimilarity was used for sample-level abundance-based comparisons, whereas Jaccard dissimilarity was used for genome- or species-level presence–absence analyses. PCoA was used to visualize CF compositional patterns at the genome and sample levels, and genome-level clustering was performed using PAM. Differences in CF-gene richness, Shannon diversity, prevalence, and cluster-level relative abundance across groups were assessed using the Kruskal–Wallis test. PERMANOVA with 999 permutations was used for analyses in which the corresponding effect sizes and significance values are explicitly reported, including the host-species CF comparison and the gastrointestinal-segment KEGG comparison. For the cross-subfamily comparison, genome-level differences in CF-family richness between Cervinae and Caprinae were evaluated using the Wilcoxon rank-sum test, and differences in CF-family prevalence between the two subfamilies were assessed using Fisher’s exact test with Benjamini–Hochberg correction where applicable. Associations between CF families and cluster assignments were evaluated using chi-squared tests with Cramér’s V as the effect-size measure. Spearman correlation analysis with Benjamini–Hochberg correction was used where applicable to assess associations between functional categories and CF organization. The association between bacterial patristic distance and CF compositional dissimilarity was summarized using Spearman’s rank correlation. For visualization, a random 0.01% subset of pairwise comparisons was displayed.

## Results

### Genome-resolved phylogenomic structure of cervid-associated microbiomes

Starting from the 15,494 MAGs previously generated from 688 cervid-associated samples, quality filtering retained 4,995 high-quality MAGs (completeness ≥ 90% and contamination ≤ 5%), which were dereplicated at 99% average nucleotide identity to yield 3,311 non-redundant MAGs for downstream analyses. Detailed genome quality and assembly metrics are provided in Supplementary Table 4.

**FIGURE 1:**
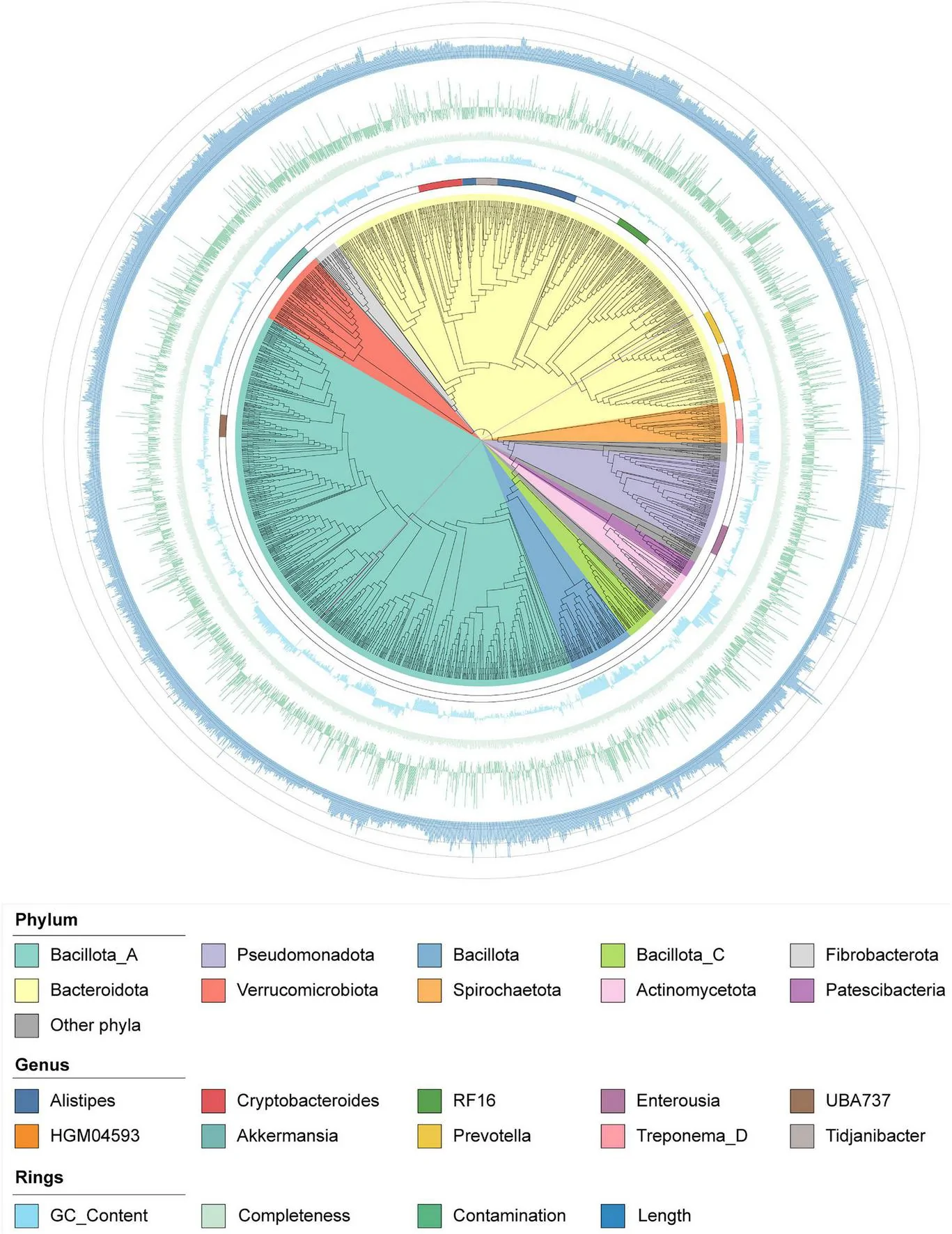
Phylogenetic tree and genome characteristics of 3,311 cervid-associated MAGs. The branches of the phylogenetic tree are color-coded at the phylum level, showing the top ten phyla with the highest numbers of MAGs, while the remaining phyla are shown in gray. The first outer ring represents genus-level classification, with colors indicating genus categories, displaying only the genera with the largest numbers of MAGs. The second, third, and fourth outer rings correspond to GC content, completeness, and contamination rate. The fifth outer ring displays a bar chart representing the genome size of each MAG.

A phylogenomic tree reconstructed from the predicted protein sequences of the 3,311 non-redundant MAGs using PhyloPhlAn3 revealed clear lineage-level organization across the dataset (Figure 1 and Supplementary Table 5). The reconstructed MAGs were predominantly assigned to Bacillota_A and Bacteroidota, which together accounted for over 70% of the dataset, while additional phyla including Pseudomonadota, Verrucomicrobiota, Bacillota_C, Actinomycetota, Spirochaetota, Fibrobacterota, and Patescibacteria were also represented. At the genus level, the community encompassed diverse lineages, with *Alistipes*, HGM04593, *Cryptobacteroides*, *Akkermansia*, RF16, *Prevotella*, *Enterousia*, *Treponema_D*, UBA737, and *Tidjanibacter* among the most prevalent taxa.

### Genome-resolved cataloging and taxonomic structuring of CF repertoires in cervid-associated microbiomes

**FIGURE 2:**
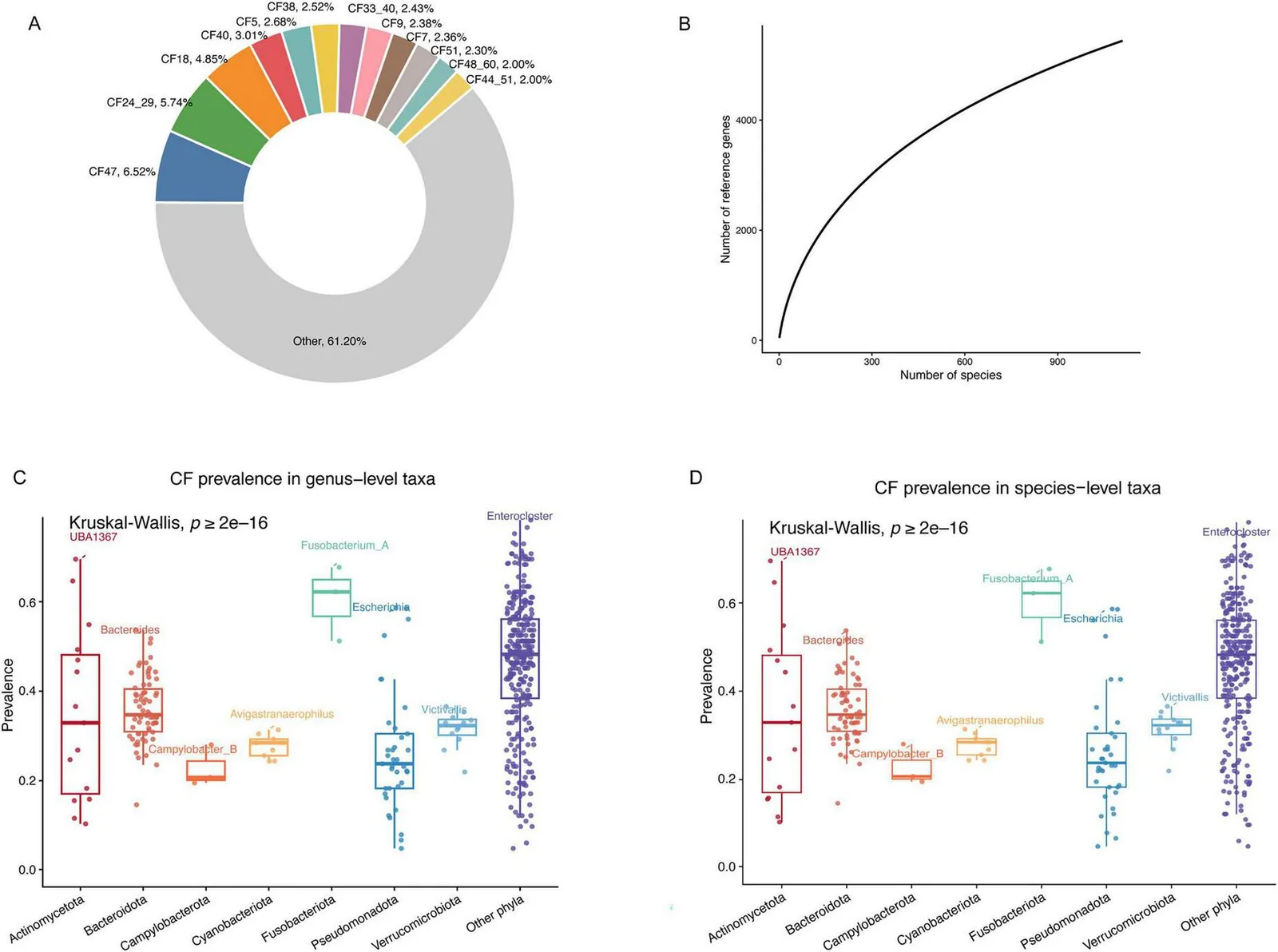
Genome-resolved characterization of CF repertoires in cervid-associated microbiomes. **(A)** Distribution of CF families detected across the non-redundant MAGs. The donut chart shows the relative proportions of the most prevalent CF families identified based on homology to the GHA reference database, with the remaining families grouped as “Other.” **(B)** Accumulation curve showing the number of detected CF reference genes as additional host species were incorporated into the analysis. **(C,D)** Taxonomic variation in CF prevalence across bacterial lineages at the genus **(C)** and species **(D)** levels. Each point represents one genus in panel **(C)** and one species in panel **(D)**. Boxplots summarize the distributions of taxon-level CF prevalence within each phylum. Differences among phylum-level groups were assessed using the Kruskal-Wallis test.

A total of 138,729 CF-associated protein-coding genes were identified across the reconstructed MAGs and classified into 71 CF families (including subfamily variants) based on homology to the GHA reference database (Supplementary Table 5). The detected repertoire was dominated by a subset of widely distributed families, among which CF47 (Peptidase_C69), CF24_29 (QueH), and CF18 (Glycos_transf_2) were the most prevalent (Figure 2A). Rarefaction analysis indicated that CF discovery had not reached saturation, implying that additional CF diversity remains to be characterized in cervid-associated microbiomes (Figure 2B).

Colonization factor prevalence showed pronounced taxonomic structuring across bacterial lineages. At both the genus and species levels, taxon-level CF prevalence differed markedly among bacterial phyla. Genera and species assigned to Bacillota_A and Bacillota generally exhibited higher and more variable CF prevalence, whereas taxa assigned to Campylobacterota and Pseudomonadota displayed lower and more constrained repertoires (Figures 2C,D). These differences were significant among phylum-level groups (Kruskal–Wallis test, *P* < 2.2 × 10^–16^).

### Cross-subfamily comparison of CF repertoires in Cervinae and Caprinae MAGs

**FIGURE 3:**
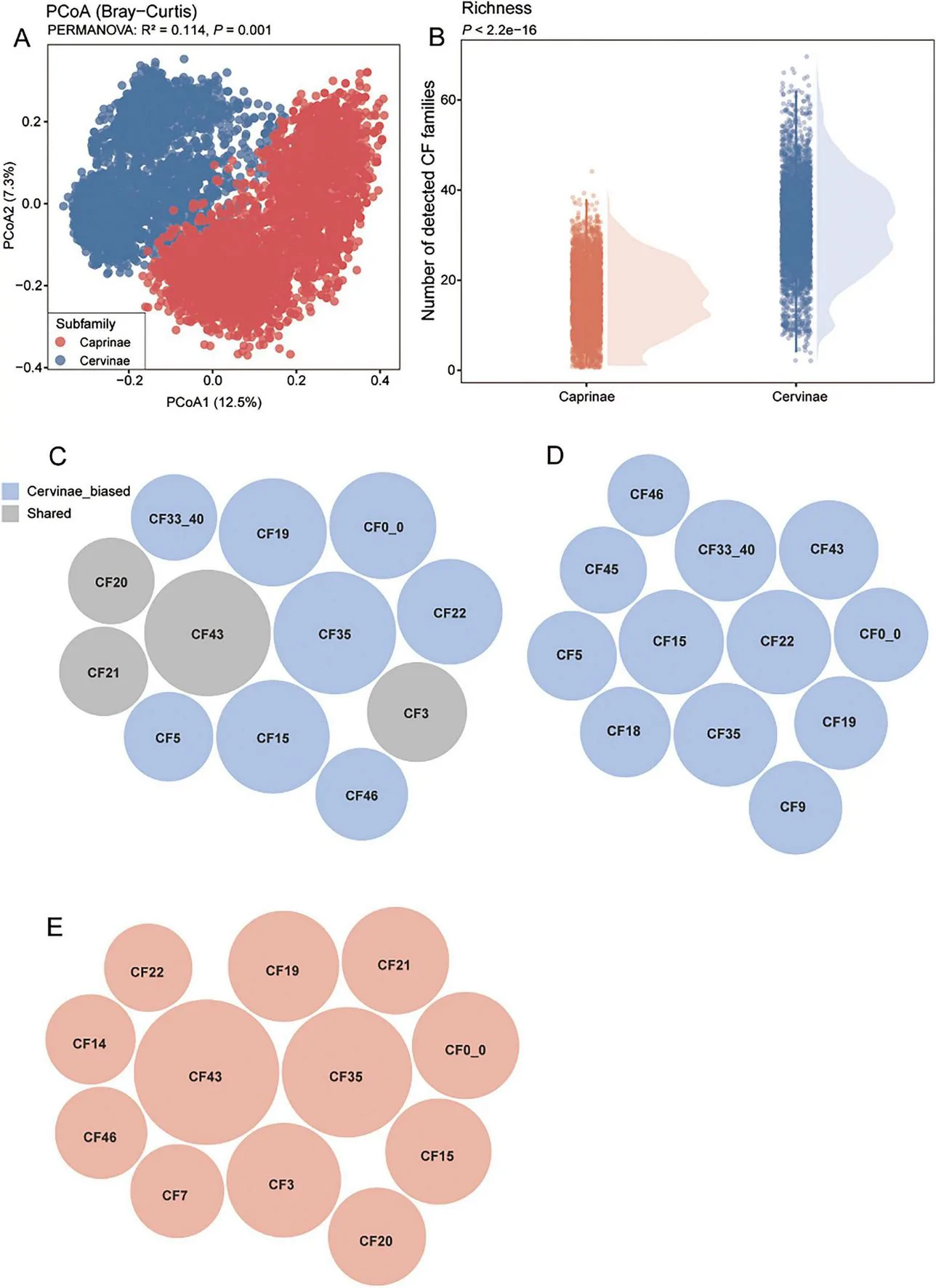
Cross-subfamily comparison of CF repertoires in Cervinae and Caprinae MAGs. **(A)** Jaccard PCoA of CF family composition in Cervinae and Caprinae genomes. Each point represents one genome. **(B)** Raincloud plot showing CF family richness per genome in Cervinae and Caprinae. **(C)** Combined overview of the top prevalent CF families ranked by mean prevalence across the two subfamilies. **(D)** Prevalence structure of the top CF families in Cervinae. **(E)** Prevalence structure of the top CF families in Caprinae. Bubble area is proportional to within-subfamily prevalence.

To place cervid CF repertoires in a broader ruminant context, we compared independently processed MAG sets from Cervinae and Caprinae. At the CF-family level, Jaccard ordination showed clear separation between the two datasets (PERMANOVA, R^2^ = 0.114, *P* = 0.001; Figure 3A). Genome-level richness analysis further showed that MAGs from Cervinae contained significantly more detected CF families than those from Caprinae (Wilcoxon, *P* < 2.2 × 10^–16^; Figure 3B and Supplementary Table 6).

Despite the overall separation between the two datasets, Cervinae and Caprinae shared a core set of highly prevalent CF families, including CF43, CF35, CF15, CF19, and CF3 (Figure 3C). Beyond this shared component, the prevalence patterns of additional CF families differed between the two groups. In Cervinae, highly prevalent families included CF15, CF22, CF35, CF33_40, CF43, and CF0_0, together with several additional families such as CF18, CF5, and CF45 (Figure 3D). In Caprinae, the most prevalent families included CF43, CF35, CF3, CF19, CF21, and CF0_0, with comparatively fewer families extending beyond this dominant set (Figure 3E).

### Taxonomic structuring and clustering of CF repertoires

**FIGURE 4:**
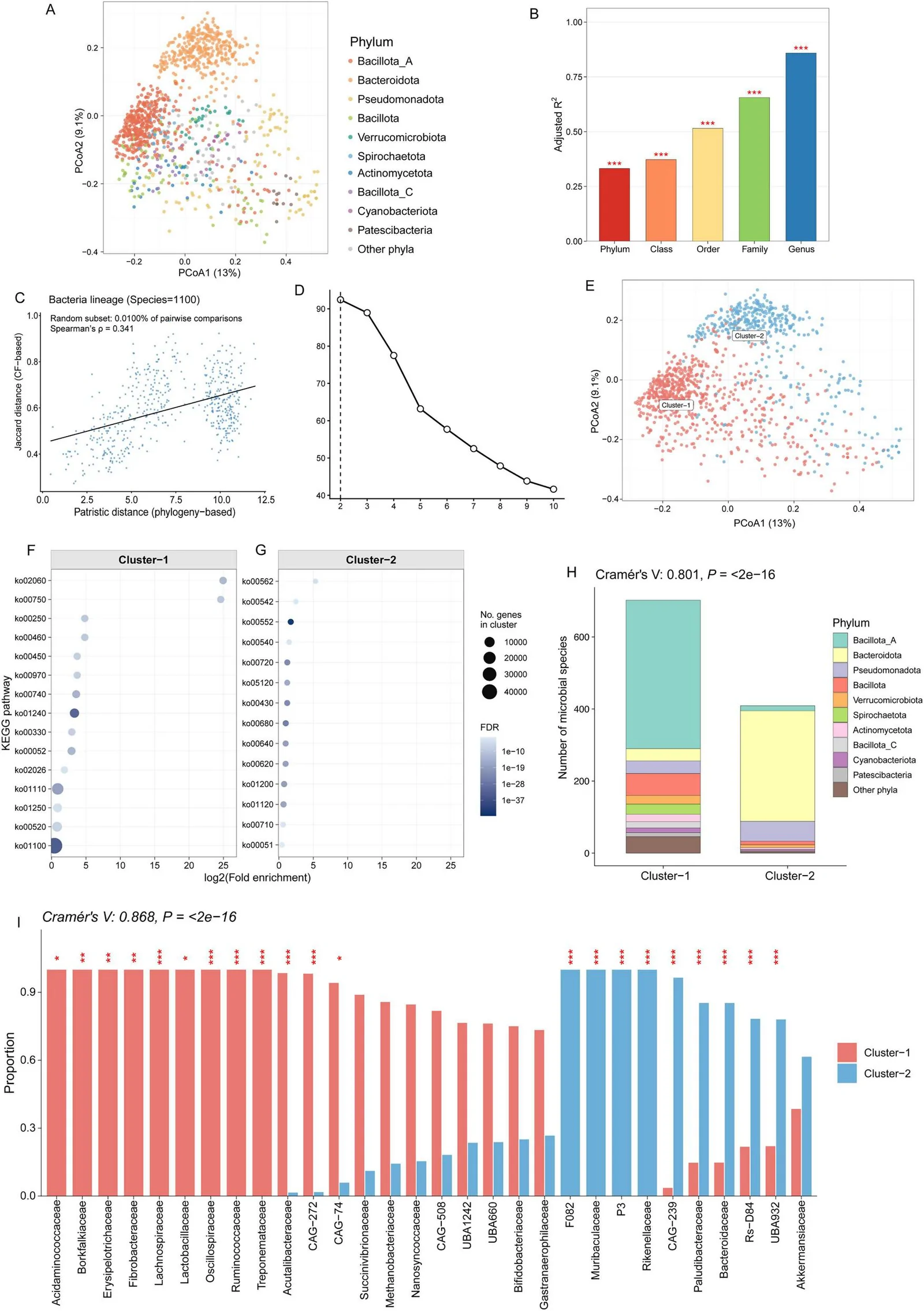
Phylogenetic structuring and two-cluster organization of CF repertoires across microbial genomes. **(A)** PCoA of bacterial species based on Jaccard dissimilarity of species-level CF repertoires, with points colored by phylum. **(B)** Proportion of CF compositional variation explained by taxonomic rank from phylum to genus, summarized as adjusted *R*^2^. **(C)** Relationship between phylogenetic distance (patristic distance) and CF compositional dissimilarity across bacterial species. **(D)** Selection of the optimal number of CF-based clusters using PAM across candidate *k*-values, with the Calinski–Harabasz index supporting two clusters. **(E)** PCoA of microbial genomes colored by CF-defined cluster membership. **(F,G)** KEGG pathway enrichment analysis for Cluster 1 **(F)** and Cluster 2 **(G)**. Dot size indicates the number of genes assigned to each pathway within the corresponding cluster, and color indicates FDR. **(H)** Phylum-level taxonomic composition of species within the two CF-defined clusters. **(I)** Family-level distribution of dominant microbial families across the two CF-defined clusters. Statistical significance is indicated as follows: *P < 0.05, **P < 0.01, and ***P < 0.001.

Colonization factor repertoires were strongly structured by microbial taxonomy and showed a moderate positive association with bacterial phylogenetic distance. Species-level CF profiles separated according to major taxonomic lineages, and the proportion of CF variation explained by taxonomy increased progressively from phylum to genus level (Figures 4A,B). CF compositional dissimilarity showed a moderate positive association with patristic distance among bacterial species (Spearman’s ρ = 0.341; Figure 4C).

PAM clustering further partitioned the genome-level CF profiles into two major groups (Figures 4D,E and Supplementary Table 7). These clusters differed not only in CF composition but also in their inferred functional potential. Cluster 1 was mainly associated with pathways related to nutrient uptake, amino acid and cofactor metabolism, PTS-mediated carbohydrate utilization, transport systems, and biofilm formation, whereas Cluster 2 showed stronger enrichment of pathways linked to carbon and energy metabolism, cell-envelope biosynthesis, and stress-associated metabolic adaptation (Figures 4F,G). These results identify two recurrent CF repertoire configurations, in contrast to the three-cluster solution previously reported for the human gut reference dataset.

The two CF-defined clusters were also taxonomically structured. Cluster membership was strongly associated with phylum-level composition (Cramér’s *V* = 0.801, *P* < 2 × 10^–16^; Figure 4H) and family-level composition (Cramér’s *V* = 0.868, *P* < 2 × 10^–16^; Figure 4I). Bacillota_A-dominated lineages were mainly assigned to Cluster 1, whereas Bacteroidota-associated lineages were concentrated in Cluster 2. At the family level, several Bacillota-associated families were preferentially assigned to Cluster 1, while Bacteroidota-associated families were more frequently assigned to Cluster 2. Together, these results show that CF repertoires in cervid gut microbiomes are taxonomically structured, moderately associated with bacterial phylogenetic distance, and organized into two recurrent configurations with distinct taxonomic and KEGG functional profiles.

### Host species-associated ecological differentiation of CF repertoires

**FIGURE 5:**
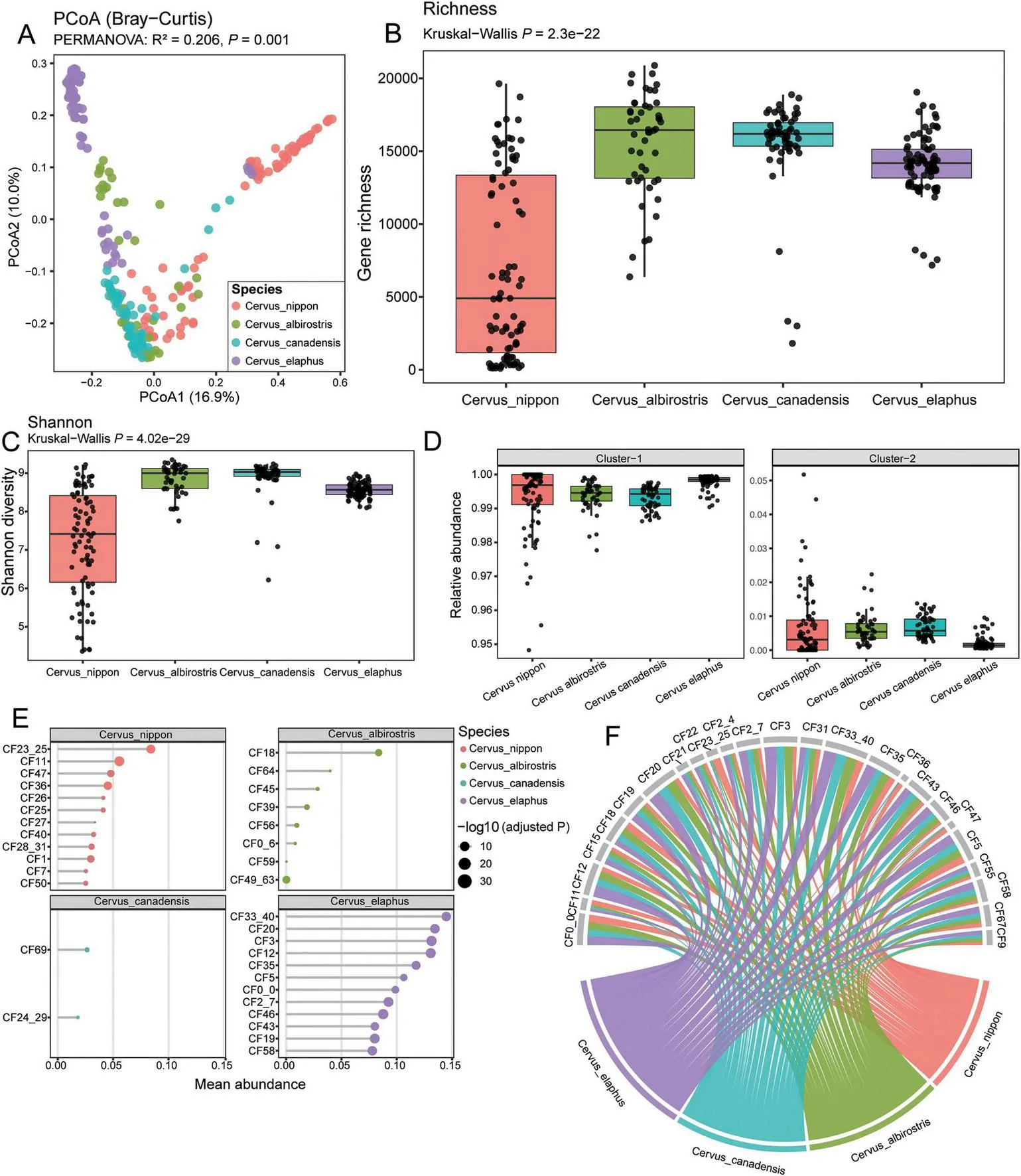
Host-species-associated differentiation of CF repertoires. **(A)** Principal coordinate analysis (PCoA) based on Bray–Curtis dissimilarities of CF-gene abundance profiles across the four host species. Each point represents one sample. Differences in CF-gene composition among host species were assessed using PERMANOVA with 999 permutations. **(B)** CF-gene richness and **(C)** Shannon diversity across host species. Each point represents one sample, and overall differences were assessed using the Kruskal–Wallis test. **(D)** Relative abundance of the two CF-defined clusters across host species. Each point represents one sample, and differences among host species were assessed using the Kruskal–Wallis test. **(E)** Dot plot showing representative CF families associated with individual host species. The *x*-axis indicates mean abundance, point color denotes host species, and point size is proportional to –log_10_ (adjusted *P*). **(F)** Chord diagram summarizing associations between host species and representative CF families. Chord width is proportional to the mean abundance of the corresponding CF family in each host species.

Colonization factor repertoires differed significantly among the four cervine host species at multiple levels. At the gene level, Bray–Curtis ordination showed clear host-species-associated separation, indicating that CF-gene composition varied systematically among *Cervus nippon*, *Cervus albirostris*, *Cervus canadensis*, and *Cervus elaphus* (PERMANOVA, R^2^ = 0.206, *P* = 0.001; Figure 5A). Alpha-diversity analyses further showed strong interspecies differences in both CF-gene richness and Shannon diversity (Kruskal–Wallis, *P* < 0.001; Figures 5B,C), indicating that host-associated variation involved both the number and relative distribution of CF-associated genes.

Host-species differences were also evident at the cluster level. Cluster 1 dominated the CF repertoire across all four host species, whereas Cluster 2 contributed only a minor fraction. Nevertheless, the relative abundances of the two clusters still varied among host species, with *Cervus elaphus* showing the highest proportion of Cluster 1 and the lowest proportion of Cluster 2 (Figure 5D). Beyond these global patterns, host-associated differentiation was further reflected at the CF-family level. Several dominant CF families showed preferential enrichment in individual host species, and these enriched families differed in both identity and relative abundance across hosts (Figure 5E). A chord diagram summarizing host-CF family associations further showed that the four host species were linked to partially distinct CF family assemblages rather than sharing a uniform repertoire structure (Figure 5F). Together, these results show that host-species-associated variation is evident not only in overall CF composition and diversity, but also in the family-level and higher-order organization of CF repertoires. A BUSCO-based host phylogeny of the four focal *Cervus* species, with *Moschus berezovskii*, *Bos taurus*, and *Capra hircus* included as outgroup taxa (Supplementary Figure 1), provides an evolutionary context for these comparisons and indicates that the observed CF divergence occurred within a relatively constrained *Cervus* host background.

### Segment-associated differentiation of CF repertoires across the gastrointestinal tract

**FIGURE 6:**
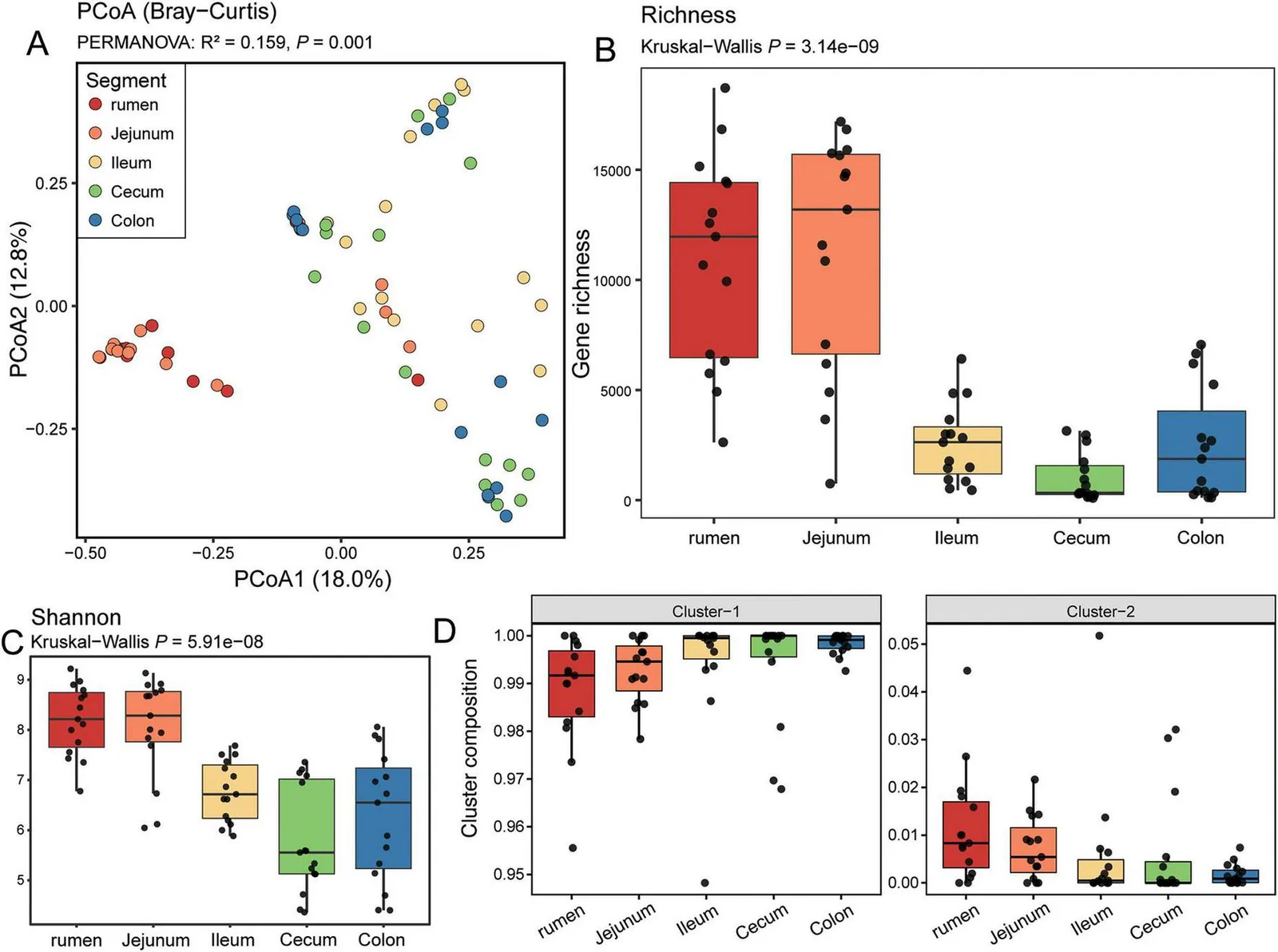
Segment-associated differentiation of CF repertoires across the gastrointestinal tract. **(A)** Principal coordinate analysis based on Bray–Curtis dissimilarities of CF-gene abundance profiles across the rumen, jejunum, ileum, cecum, and colon. Each point represents one sample. **(B)** CF-gene richness and **(C)** Shannon diversity across gastrointestinal segments. Each point represents one sample, and overall differences were assessed using the Kruskal–Wallis test. **(D)** Relative abundance of the two CF-defined clusters across gastrointestinal segments. Each point represents one sample.

Colonization factor repertoires varied across gastrointestinal segments at both the composition and diversity levels. At the gene level, Bray–Curtis ordination revealed segment-associated variation in CF-gene composition along the gastrointestinal tract (Figure 6A). Alpha-diversity analysis further showed that CF-gene richness and Shannon diversity differed significantly among segments (Kruskal–Wallis, *P* < 0.001 for both; Figures 6B,C). Jejunal and ruminal samples showed the highest CF-gene richness, whereas cecal samples showed the lowest values, with ileal and colonic samples occupying intermediate positions. A similar pattern was observed for Shannon diversity, supporting marked segment-associated differences in both the presence and relative distribution of CF-associated genes.

This segment effect was also evident at the cluster level. Across all gastrointestinal segments, Cluster 1 accounted for the vast majority of CF abundance, whereas Cluster 2 remained consistently low (Figure 6D). Nonetheless, the relative abundances of the two clusters varied across segments, with Cluster 2 showing higher abundance in the rumen and jejunum and lower abundance in the ileum, cecum, and colon; the opposite pattern was observed for Cluster 1.

### Segment-associated KEGG functional stratification provides additional context for CF differentiation

**FIGURE 7:**
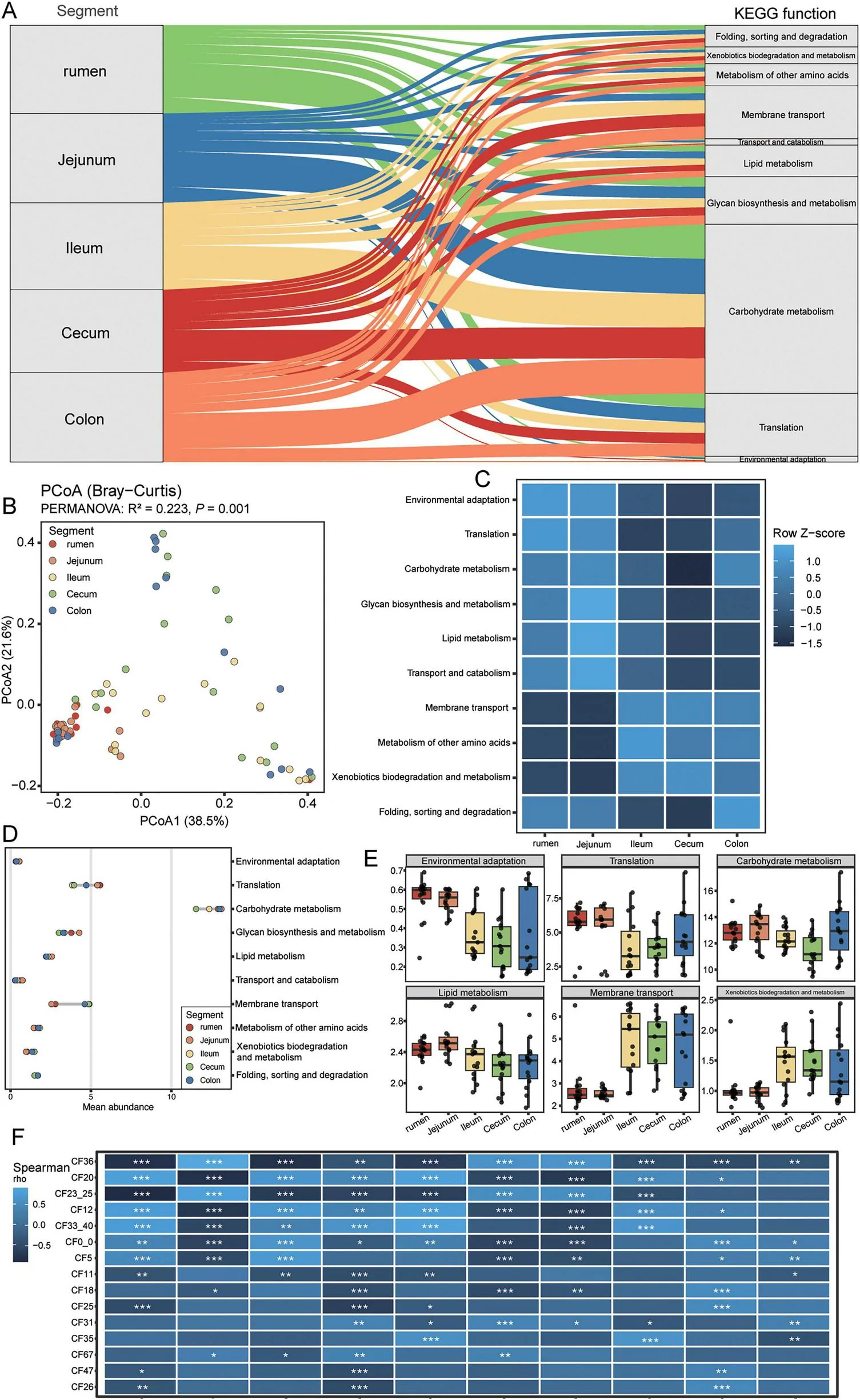
Segment-associated KEGG functional stratification and its alignment with CF organization. **(A)** Alluvial plot showing the distribution of 10 selected KEGG B-level categories across the five gastrointestinal segments. Flows connect each segment to the displayed KEGG categories, and flow width is proportional to the mean abundance of each category within the corresponding segment. **(B)** Principal coordinate analysis based on Bray–Curtis dissimilarities of KEGG functional profiles across gastrointestinal segments. Each point represents one sample. Differences in overall KEGG composition among segments were assessed using PERMANOVA with 999 permutations. **(C)** Heatmap showing segment-wise abundance patterns of the displayed KEGG B-level categories. **(D)** Cleveland dot plot summarizing the mean abundance of the displayed KEGG categories in each segment. **(E)** Boxplots showing the distributions of representative KEGG categories across gastrointestinal segments. Each point represents one sample. **(F)** Spearman correlation heatmap showing associations between the abundances of the displayed CF families and KEGG B-level categories across the 75 samples. *P*-values were adjusted using the Benjamini–Hochberg procedure. Significance levels are indicated as follows: *adjusted *P* < 0.05, **adjusted *P* < 0.01, and ***adjusted *P* < 0.001. The KEGG categories shown in panels **(C–F)** represent a subset displayed to summarize the major segment-associated functional patterns; complete KO-level functional profiles for all 75 samples are provided in Supplementary Table 8.

To further characterize the functional background underlying the segment-associated differences in CF composition described above, KEGG profiles were compared across the same five gastrointestinal regions. Complete KO-level functional profiles for all 75 samples are provided in Supplementary Table 8. At the overall level, KEGG categories were redistributed across gut segments rather than remaining uniformly represented along the intestinal tract (Figure 7A). This pattern was supported by ordination analysis, which showed clear separation of segment-specific KEGG profiles in Bray–Curtis space (PERMANOVA, R^2^ = 0.223, *P* = 0.001; Figure 7B). The segment effect was also reflected at the level of individual functional categories. Jejunal communities showed relatively higher representation of carbohydrate metabolism, glycan biosynthesis and metabolism, and lipid metabolism, whereas ileal samples were characterized more strongly by membrane transport. Cecal samples showed stronger xenobiotics biodegradation and metabolism, while ruminal communities were distinguished by higher environmental adaptation and translation. Across these categories, colonic samples generally occupied an intermediate position rather than showing a dominant enrichment pattern comparable to that of the jejunum, ileum, cecum, or rumen (Figures 7C–E). Together, these results indicate that different gastrointestinal regions are associated with distinct KEGG functional states, providing an additional layer of segment-associated structure beyond the CF differences themselves.

We next examined correlations between CF family abundances and the KEGG categories that varied across gastrointestinal segments. Multiple dominant CF families showed significant positive or negative correlations with these categories (Figure 7F), indicating covariation between CF composition and broader functional profiles across segments.

## Discussion

Genome-resolved analyses of CF repertoires remain scarce in mammalian gut microbiome research (Kim et al., 2024). Existing work has more often focused on individual adhesion-associated genes, specific colonization determinants, taxonomic composition, or the genomic characterization of antimicrobial resistance and virulence in animal-associated bacterial isolates (Liu et al., 2026c; Ni et al., 2026; Powell et al., 2016; Xiao et al., 2021; Yang et al., 2026), leaving broader patterns of CF organization across genomes, hosts, and gut environments less well defined. By integrating multiple publicly available cervid metagenomic projects together with an in-house dataset, we examined CF repertoires within a comparative genome-resolved framework, with particular attention to taxonomic structuring, host-associated differentiation, and gastrointestinal spatial heterogeneity. We therefore interpreted the observed patterns within a hierarchical framework in which microbial taxonomy and phylogenetic relatedness are associated with genome-level CF organization, whereas host species and gastrointestinal segment contribute to ecological differentiation, together producing ecological stratification across cervid gut microbiomes.

A central result of this study is that CF repertoires were strongly structured by microbial taxonomy rather than being diffusely distributed across genomes. CF prevalence differed markedly among higher bacterial lineages, the proportion of explained variation increased toward lower taxonomic ranks, and CF compositional dissimilarity showed a moderate positive association with bacterial phylogenetic distance. Together, these patterns suggest that CF repertoires likely follow a broader rule of genome organization in which ecologically relevant functions are shaped jointly by evolutionary history and host-associated selection (Brown et al., 2023; Groussin et al., 2017; Rühlemann et al., 2024).

The Cervinae–Caprinae comparison was included to place cervid CF repertoires within a broader ruminant context, rather than to test subfamily-level evolutionary divergence. Previous metagenomic characterization of Caprinae gut microbiota has focused on antimicrobial resistance, virulence, and mobile genetic elements (Su et al., 2026), whereas the present comparison extends this genomic perspective to CF repertoires. The observed separation in CF composition and the higher CF-family richness of the Cervinae-derived MAG set therefore provide a comparative benchmark for interpreting the cervid-focused patterns identified here. The shared prevalence of several dominant CF families may represent a common component of CF organization across the two ruminant-derived MAG sets, whereas differences in additional families contributed to their separation. However, differences between the two metagenome-derived genome sets may also reflect variation in host ecology and the taxonomic structure of the underlying microbial communities (Youngblut et al., 2019), as well as differences in sampling design, project composition, and sample-processing procedures (Costea et al., 2017). Accordingly, these contrasts should not be attributed solely to evolutionary divergence or subfamily-specific adaptation, but should instead be interpreted as broader comparative context for cervid CF organization.

At a finer level of organization, the two recurrent CF repertoire configurations indicate that variation in CF content is structured rather than randomly distributed across microbial genomes. Cluster membership was associated with both taxonomic composition and distinct KEGG enrichment profiles, indicating that the two configurations occurred within different genomic and functional backgrounds. Similar genome-level trait clustering has been reported in comparative studies of microbial genomes, in which interaction- or niche-related traits recur in non-random functional groupings (Krause et al., 2014; Martiny et al., 2015; Zoccarato et al., 2022). However, these associations do not establish experimentally validated colonization strategies or adaptive programs; rather, they support a repertoire-level framework in which CF organization is linked to broader taxonomic and functional structure. This taxonomic organization and moderate phylogenetic association provide the background against which host-associated CF differentiation should be interpreted.

Host-species-associated differences should be interpreted in the context of the strong microbial lineage dependence described above. Because CF repertoires were associated with microbial taxonomy and bacterial phylogenetic distance, and the two recurrent configurations were taxonomically structured, variation among the four *Cervus* hosts may have arisen partly through host-associated turnover in the relative representation of microbial lineages carrying different CF repertoires. Host identity and microbial lineage composition therefore are unlikely to act as fully independent influences; rather, host-associated ecological filtering may reweight a taxonomically structured pool of microbial lineages and their associated CF configurations. The significant differences in cluster-level relative abundance among hosts are consistent with this interpretation, although the present analysis does not formally partition the respective contributions of host effects and microbial taxonomic turnover.

Among the four cervine hosts, *Cervus albirostris* may provide the most interpretable ecological case, because it is largely restricted to the Tibetan Plateau. Previous metagenomic and genome-resolved studies in plateau-adapted cervids and Tibetan antelope have pointed to broader microbiome remodeling involving energy metabolism, vitamin biosynthesis, bile acid-related functions, and other genome-scale functional features, including signals involving *Alistipes* and *Faecousia*, which were also prevalent in our dataset (Li et al., 2025; Liu et al., 2026a,b; Yu et al., 2026). More broadly, studies of high-altitude ruminants and other wild mammal systems have likewise shown that gut microbiota can undergo function-level remodeling involving energy harvesting, plant biomass degradation, and vitamin-associated pathways under environmental or biotic pressure (Ma et al., 2019; Zhang et al., 2016; Zhang X. et al., 2026). The co-occurrence of genus-level energy metabolism enrichment and CF-level compositional differentiation in plateau-associated hosts therefore suggests that persistence-related functions and broader metabolic adaptation may respond to some of the same environmental pressures, a possibility that will require direct testing in future controlled comparisons.

Gastrointestinal segments differed markedly in CF-gene richness and cluster-level composition, with the highest richness observed in the rumen and jejunum and the lowest in the cecum. Cluster 1-associated CF families accounted for most of the CF abundance across all segments, whereas the relative contribution of Cluster 2 was higher in the rumen and jejunum than in the ileum, cecum, and colon (Mizrahi et al., 2021). These abundance patterns were accompanied by segment-associated differences in broader KEGG profiles, including greater representation of environmental adaptation and translation in the rumen and of carbohydrate metabolism, glycan biosynthesis and metabolism, and lipid metabolism in the jejunum (Bevins and Salzman, 2011). The rumen and jejunum differ substantially in digestive function and local metabolic conditions, providing a plausible ecological context for these associations (Flint et al., 2012; Hillman et al., 2017). However, the observed CF and KEGG patterns should be interpreted as co-occurring features of distinct gastrointestinal environments rather than evidence that a shared cluster configuration confers environmental responsiveness or that either pattern directly drives the other.

The cecum showed a distinct segment-associated pattern, with the lowest CF richness and relatively greater representation of xenobiotics biodegradation-related functions. This co-occurrence is consistent with the possibility of stronger ecological filtering in the cecum, but does not demonstrate that detoxification-related functions dominate the local environment or directly shape CF repertoire structure. Forage-derived plant secondary metabolites (PSMs) reach the hindgut in their most recalcitrant forms after upstream rumen and small-intestinal processing (Tan et al., 2024), and cecal communities in other cervid and ruminant systems have been specifically implicated in this detoxification role. In reindeer, dietary lichen – rich in antimicrobial phenolic PSMs – induced distinct microbial shifts in the cecum that were not mirrored in the rumen, with cecal communities responding selectively to PSM-associated compounds that escaped foregut fermentation (Salgado-Flores et al., 2016). Microbial mediation of dietary plant toxin tolerance through gut microbiota has also been demonstrated experimentally in mammalian herbivores, with evidence that the capacity to process PSMs is encoded within the gut microbial community rather than the host alone (Kohl et al., 2014). Accordingly, the comparatively narrow CF repertoire observed in the cecum should be interpreted as a plausible ecological association with its xenobiotic-related functional context, rather than as evidence of a causal relationship. Taken together, the segment-level CF and KEGG patterns indicate that colonization-associated repertoire configurations vary across gastrointestinal environments and are correlated with local functional profiles rather than forming a uniform spatial pattern.

Although a unified analytical workflow was applied, the host-species comparison integrated samples from different projects and may therefore have been influenced by geographic origin, captivity status, study design, and other project-specific factors. Accordingly, the observed differences should be interpreted as species-associated patterns rather than effects attributable solely to host identity. More broadly, although this study provides a genome-resolved, system-wide characterization of colonization factor organization across cervid gut microbiomes, the current CF catalog remains incomplete, and the links between CF repertoires and segment-associated functional profiles are correlational. Future work should prioritize expanded sampling across host species and ecological contexts, alongside mechanistic and longitudinal approaches to establish the functional consequences of the variation described here.

## Conclusion

Colonization factor repertoires in cervid gut microbiomes exhibited a hierarchical pattern of ecological stratification. At the genome level, CF organization was strongly associated with microbial taxonomy, moderately related to bacterial phylogenetic distance, and expressed through two recurrent CF configurations. At the ecological level, CF composition and diversity differentiated across host species and gastrointestinal segments, with these patterns occurring alongside segment-associated functional variation. Whether these database-defined repertoire patterns translate into differences in microbial persistence, host adaptation, or microbiome stability requires further experimental and longitudinal validation.

## Data Availability

The original contributions presented in this study are included in this article/Supplementary material, further inquiries can be directed to the corresponding authors.

## Footnotes

1
https://doi.org/10.6084/m9.figshare.31900999

## Supplementary material

Supplementary Figure 1 Host phylogeny and host-level variation in CF repertoires. Maximum-likelihood phylogeny of the four focal *Cervus* species and three outgroup ruminant taxa reconstructed from concatenated single-copy BUSCO orthologs. The four deer species (*Cervus nippon*, *Cervus elaphus*, *Cervus canadensis*, and *Cervus albirostris*) formed a well-supported cervid clade, whereas *Moschus berezovskii* was resolved outside Cervidae. *Bos taurus* and *Capra hircus* were included as additional ruminant reference lineages.
