# Supplementary material for: Genome-resolved analysis of colonization factor repertoires reveals ecological stratification in cervid gut microbiomes
Source: Front Microbiol. 2026 Aug 27;17:1929306. doi: 10.3389/fmicb.2026.1929306 (PMC13558222; doi:10.3389/fmicb.2026.1929306)
Supplement: Supplementary file 1 [file Image_1.pdf]

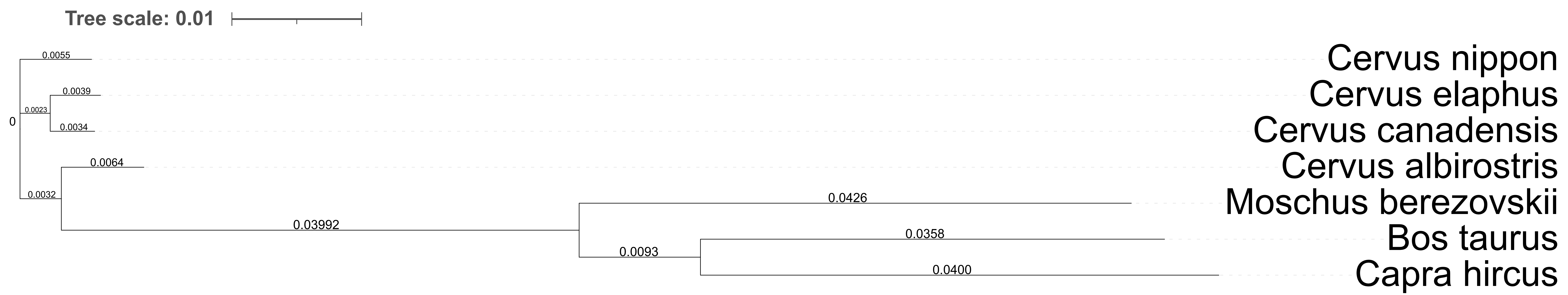

Tree scale: 0.01
0.0055
Cervus nippon
0.0039
Cervus elaphus
0.0023
0
0.0034
Cervus canadensis
0.0064
Cervus albirostris
0.0032
0.0426
Moschus berezovskii
0.03992
0.0358
Bos taurus
0.0093
0.0400
Capra hircus
